# “It is difficult to be absolutely sure one way or the other.” – a mixed method study on Finnish physicians’ views on euthanasia and physician-assisted suicide

**DOI:** 10.1186/s12910-025-01220-6

**Published:** 2025-05-21

**Authors:** Reetta P. Piili, Minna Hökkä, Elina Tolvanen, Jukka Vänskä, Pekka Louhiala, Juho T. Lehto

**Affiliations:** 1 Faculty of Medicine and Health Technology, Tampere University, Tampere, Finland; 2 Palliative Care Centre, Tampere University Hospital, Tampere, Finland; 3 Diaconia University of Applied Sciences, Helsinki, Finland; 4 Finnish Medical Association, Helsinki, Finland; 5 Faculty of Social Sciences, Tampere University, Tampere, Finland; 6 Clinicum, Faculty of Medicine, University of Helsinki, Helsinki, Finland

**Keywords:** Assisted dying, Euthanasia, Physician-assisted suicide, Physician, Ethics

## Abstract

**Background:**

Euthanasia and physician-assisted suicide (PAS) are complex and ethically challenging topics. Physicians’ attitudes toward euthanasia and PAS have been studied, but little is known about their ethical considerations regarding these topics. This study aimed to assess Finnish physicians’ views on assisted dying (AD), including euthanasia and PAS. Our special emphasis was to describe physicians’ views on the legalization of AD, their views on AD as a phenomenon, and how AD reflects on physicians’ roles.

**Methods:**

A survey including statements and open questions concerning euthanasia and PAS was sent to all Finnish physicians in 2020. The data was analysed using quantitative measures and a qualitative approach.

**Results:**

Altogether, 6889 physicians answered the survey, yielding a response rate of 26%. Of the responders, 9% fully agreed that accepting euthanasia would benefit the physician-patient relationship, while 19% fully agreed that it would harm this relationship. From 2565 responders, 3033 answers were received to the open questions. The qualitative analysis yielded two unifying categories, firstly ‘Physicians’ views on assisted dying (AD) in the current societal situation and on its legalization’ included three main categories namely: ‘AD and end-of-life issues in the current societal situation’ (f = 230), ‘Physicians perspectives on legalisation of AD’ (f = 605) and ‘The possible consequences of legalizing AD’ (f = 543). Secondly, the unifying category ‘Physicians views on AD as part of their work and as a phenomenon’ included two main categories, ‘AD and the physicians’ professional role (f = 650) and ‘AD as a multifaceted phenomenon’ (f = 296).

**Conclusion:**

Assisted dying is seen as a complex issue, and it was difficult to form an opinion on it. Physicians and the care team are faced with ethical dilemmas about topics related to AD, even though it is not legalized in Finland. Open and pluralistic discussion on AD, including ethical aspects, implications for society and end-of-life care practices is of utmost importance.

**Supplementary Information:**

The online version contains supplementary material available at 10.1186/s12910-025-01220-6.

## Introduction

Discussions concerning different aspects of assisted dying (AD), including euthanasia and physician-assisted suicide (PAS), are ongoing around the world. Cultural, political, clinical, legal, and ethical considerations need to be taken into account when having these discussions.

While practices of euthanasia and PAS are adopted in quite many Western countries, they are still a marginal phenomenon globally [1–3]. The World Medical Association (WMA) considers euthanasia to be unethical [4]. Although general support for euthanasia and assisted suicide has been rising [5]. The Finnish Medical Association is in line with the WMA and objects to the legalization of euthanasia [6]. The International Association for Hospice and Palliative Care (IAHPC) and the European Association for Palliative Care (EAPC) have stated, in 2017 and 2016, respectively, that euthanasia and PAS should not be included as part of the clinical practice of palliative care [7, 8]. However, the British Medical Association recently adopted a neutral position on physician-assisted dying, and the Royal Dutch Medical Association is in line with local legislation, according to which physicians are allowed to perform euthanasia and PAS [9, 10].

Considering ethical issues in healthcare and end-of-life care, the four main principles are: non-maleficence (do no harm), beneficence (do good), autonomy (the right of self-determination) and justice (e.g. appropriate use and allocation of healthcare resources) [11, 12]. Others include, for example, truth-telling, confidentiality and the professional-patient relationship. Respecting life can be considered one of the fundamental principles of medicine [11, 12]. This has been one of the arguments objecting legalization of euthanasia and PAS. In the Charter of Fundamental Rights of the European Union, human dignity is called out in the first article, and in Finland, the National Advisory Board on Social Welfare and Health Care Ethics (ETENE) raised the term human dignity as an important ethical principle, especially in end-of-life situations [13, 14]. Human dignity has been used as an argument both for and against euthanasia and assisted suicide.

Physicians’ support for euthanasia and PAS has been shown to be lower than among the public [5]. Overall, in countries where AD is allowed, the support among physicians is high, whereas in countries where it is not allowed, the support remains lower [5]. However, the variation of opinions among physicians is large [5]. In Finland, there is a clear division of the opinions on AD [15]. This division of opinions highlights that ethical dilemmas around euthanasia and PAS are complex and contentious.

In countries where AD is performed, physicians have had mixed feelings after taking part in AD, ranging from moral distress to feelings of success in alleviating suffering [16–21]. Medical assistance in dying (AD in Canada) has also enriched the capacity for caring and altered the relationship with patients and families, according to a Canadian study [22].

Euthanasia is not legalized or decriminalized in Finland, and although euthanasia is not specifically mentioned in law, it is considered as a criminal act. Assistance in suicide is not specifically mentioned in Finnish law. However, the potential consequences for a physician performing PAS or euthanasia are not known since these actions have never been tested in a court of law. Neither euthanasia nor PAS has been reported to be practiced in Finland.

An initiative calling for the legalization of euthanasia was rejected by the Finnish Parliament in 2018 [23]. After that, an expert group established by the Finnish Ministry of Social Affairs and Health did not reach a common understanding on the legalization of euthanasia or assisted suicide in Finland [24]. A new citizen initiative was launched in the autumn of 2023 and was given to the parliament in May 2024 [23]. The new initiative is expected to spark debate and discussions about AD, making it crucial to gain a broad overview of physicians’ views. Similar debates are ongoing in other countries, including the United Kingdom, Ireland, and Japan [25, 26].

Many of the previous studies concerning physicians’ attitudes and opinions toward AD are quantitative [27–31]. Qualitative research is mostly done with quite a small number of participants [32–34]. A broader overview which goes beyond the numbers is needed.

This study aimed to assess Finnish physicians’ views on euthanasia and PAS with special emphasis on physicians’ views on the legalization of AD, AD as a phenomenon and how AD reflects on physicians’ roles.

## Materials and methods

This mixed methods study includes both quantitative and qualitative analysis.

### Participants

An electronic survey including statements and open questions concerning PAS and euthanasia, as well as background information, was sent to all Finnish physicians and medical students with permission to work as a physician under supervision and who were members of the Finnish Medical Association (*n* = 26,740). Those whose email addresses were not known or had denied participation in surveys were excluded. The survey was conducted in 2020 via the Finnish Medical Association.

### The survey

The survey included an introduction letter and a questionnaire. The introduction letter included information about the study, guarantee of anonymity, and voluntariness. In the questionnaire, PAS was defined as follows: a physician deliberately helping a person to commit suicide by giving drugs to the person to take them by him/herself on this person’s voluntary and competent request. Euthanasia was defined as follows: a physician deliberately killing a patient by administering drugs at the patient’s voluntary and competent request. The legal status of these actions was also mentioned in the questionnaire; Euthanasia is a punishable act under the criminal law in Finland [35]. Because suicide is not considered a criminal act, assistance in suicide is not regarded as a criminal act. However, healthcare professionals have a special obligation to protect the patients they take care of; thus, it can be assumed that the act of a physician will not remain without legal consequences.

The questionnaire included several statements concerning euthanasia and PAS. In this study, we included two statements: “Accepting euthanasia would harm the doctor-patient relationship in general” and “Accepting euthanasia would benefit the doctor-patient relationship in general”, for which results have not been previously published. The participants were asked to express their agreement on statements on a Likert scale: fully agree, partly agree, partly disagree, fully disagree, or I cannot say. In addition to statements, participants were asked if they had faced a request for euthanasia or assistance in suicide by a patient or a patient´s relative. If the participant answered yes, an open question followed: “Would you describe the situation and your actions shortly?” Another open question was also included: “If there is anything else you would like to share with the Finnish Medical Association concerning euthanasia or physician-assisted suicide, please tell us”. Additionally, some background information, such as age, gender and self-reported experience in the care of dying patients (yes or no) and for how long (not at all/less than 5 years/5–10 years/more than 10 years), were asked. No prior relationship was established between the researchers and the participants in the survey. The questionnaire is available as a supplementary file 1.

Parts of the results from this survey have been previously published [15, 36]. A study comparing parts of the quantitative data was published in 2022 [15] and another, published in 2024 [36], included previously unpublished quantitative data and qualitative data from a 2020 survey concerning physicians’ actions when facing a request for euthanasia or PAS.

### Ethical considerations

The survey was performed by the Finnish Medical Association using their member registry. The association has permission to send questionnaires to its members if they have not declined this. Responding was anonymous, and participation was voluntary. Participants gave their informed consent by voluntarily answering the questionnaire. The research data did not include any personally identifiable information. According to Finnish legislation, ethics approval is not needed in this type of questionnaire study [37]. This study was conducted according to national laws, regulations, and the Declaration of Helsinki.

### Data analysis

The answers of the responders are described with numbers and proportions. When assessing relations between background factors and the answers concerning the statements “Accepting euthanasia would harm the doctor-patient relationship in general” and “Accepting euthanasia would benefit the doctor-patient relationship in general” the Likert scale was converted into two options: fully/partly agree and fully/partly disagree/I can’t say. These two-scale answers and background factors were tested using the Pearson chi-square test. Two-sided p-values less than 0.05 were considered statistically significant. Data analysis was performed using IBM SPSS Statistics for Windows, Version 27.0 (IBM Corporation, 2020).

The qualitative analysis was performed by using an inductive content analysis approach, where categories emerge from the data, and no theoretical framework was used as a starting point as recommended in the guidelines [38, 39]. The qualitative data consisted of answers to the two open questions. After careful familiarization with the qualitative data, three research questions were posed: (1) What were physicians’ views on assisted dying and end-of-life issues in the current societal situation? (2) What were physicians’ views on the legalization of AD? (3) What were physicians’ views on assisted dying as a phenomenon, and how does this reflect their role as physicians? Only the manifest contents of the data were analyzed. The units of analysis consisted of words, sentences, or phrases that constructed a meaning.

The analysis followed four phases: (1) transcribing the data verbatim from the questionnaires to a Microsoft Word template and familiarizing with the data. No software was used in the analysis. During this phase, the research questions were refined since the data directed the analysis process [38]; (2) reducing and coding the data that were relevant to the study aim; (3) grouping the reduced expressions based on similarities; and (4) forming subcategories, categories, main categories and unifying categories of the data. The analysis process was performed by two authors who have formal education and experience in conducting qualitative research concerning this subject and other subjects as well, each phase first individually and then assessing the findings together and building consensus. After the first authors performed the coding and categorization of the data, it was critically assessed by the other authors who have experience in conducting qualitative research. The frequencies (f) of the reduced codes were counted to describe the noteworthiness of each category in relation to the entirety. The collected data were rich and yielded a total of 2326 codes. Data saturation was achieved during the analysis, which indicates that the data were sufficient [40].

## Results

The characteristics of the responders are presented in Table 1. The response rate was 26%, as 6889 physicians answered the survey. The majority of the responders were women (59%). The largest age group was physicians over 65 years old, and approximately one-fourth of the responders were retired. Of the responders, 16% had faced a request for euthanasia or assistance in a suicide from a patient or patient’s relative. At the time of the survey, 42% of the participants were involved in taking care of patients at their end-of-life. Almost one-third of all responders had over 10 years of experience in end-of-life care.

**Table 1:** Characteristics of the responders

	*n*	(%)
Number of participants	6889	
Female	4020	(59)
Age distribution		
< 35 y	1437	(21)
35–44 y	1220	(18)
45–54 y	1131	(16)
55–64 y	1292	(19)
≥ 65 y	1798	(26)
Specialty in full-time job		
Operative	1408	(21)
Conservative	1645	(24)
Diagnostic	426	(6)
Psychiatric	566	(8)
General medicine, occupational medicine, and other fields	1611	(23)
Not specialized	1218	(18)
Current working status		
Working	4656	(68)
Student	400	(6)
Retired	1626	(24)
Out of work due to another reason	191	(3)
Currently taking care of dying patients		
Yes	2859	(42)
No	3953	(57)
Amount of experience from care of patients at their end-of-life		
Not at all	1113	(16)
< 5 y	2778	(41)
5–10 y	912	(13)
> 10 y	2050	(30)
Patient or patient’s relative having ask for euthanasia or PAS		
Yes	1068	(16)
No	5821	(85)

PAS, physician-assisted suicide

**Table 2:** Agreement with the statements concerning the impact of euthanasia on doctor-patient relationship

Statement, *n* (%)		Fully agree			Partly agree			Partly disagree			Fully disagree			I can’t say	
Accepting euthanasia would harm the doctor-patient relationship		1324	(19)		1254	(18)		1323	(19)		2102	(31)		868	(13)
Accepting euthanasia would benefit the doctor-patient relationship		619	(9)		1446	(21)		863	(13)		2112	(31)		1831	(27)

The statements concerning the doctor-patient relationship are shown in Table 2, and associations between background factors and these statements are presented in Table 3. One-third of the responders fully disagreed that accepting euthanasia would harm the doctor-patient relationship. On the other hand, one out of ten responders fully agreed that accepting euthanasia would benefit the doctor-patient relationship. Older responders, responders participating and having the most experience with care of patients at their end-of-life agreed more frequently that accepting euthanasia would harm the doctor-patient relationship. Male responders and responders whose patients or patients’ relatives have asked for euthanasia or PAS agreed more often that accepting euthanasia would benefit the doctor-patient relationship.

**Table 3:** Proportion of the responders with different background factors fully or partly agreeing the statements concerning the impact of euthanasia on doctor-patient relationship

	Accepting euthanasia would harm the doctor-patient relationship		*P*-value*	Accepting euthanasia would benefit the doctor-patient relationship		*P*-value*
	Fully/Partly agree, n (%)			Fully/Partly agree, n (%)		
Gender			0.813			*< 0.001*
Female	1502	(37)		1051	(26)	
Male	1049	(38)		987	(35)	
Age			*< 0.001*			*< 0.001*
< 35 y	442	(31)		474	(33)	
35–44 y	423	(35)		375	(31)	
45–54 y	404	(36)		368	(33)	
55–64 y	529	(41)		313	(24)	
> 65 y	776	(43)		532	(30)	
Specialty in full-time job			*< 0.001*			*< 0.001*
Operative	493	(35)		477	(34)	
Conservative	704	(43)		433	(26)	
Diagnostic	138	(32)		166	(39)	
Psychiatric	207	(37)		159	(28)	
General medicine, occupational medicine, and other fields	636	(40)		425	(26)	
Not specialized	396	(33)		398	(33)	
Participates in the care of patients at their end-of-life			*< 0.005*			0.196
Yes	1124	(39)		838	(29)	
No	1422	(36)		1214	(31)	
Amount of experience			*< 0.001*			*0.010*
Not at all	325	(29)		359	(32)	
< 5 y	962	(35)		861	(31)	
5–10 y	349	(38)		279	(31)	
> 10 y	928	(46)		559	(27)	
Patient or patients’ relative having asked for euthanasia or PAS			0.075			*< 0.001*
Yes	373	(35)		421	(40)	
No	2205	(38)		1644	(28)	

* Pearson Chi-Square

**Fig. 1:**
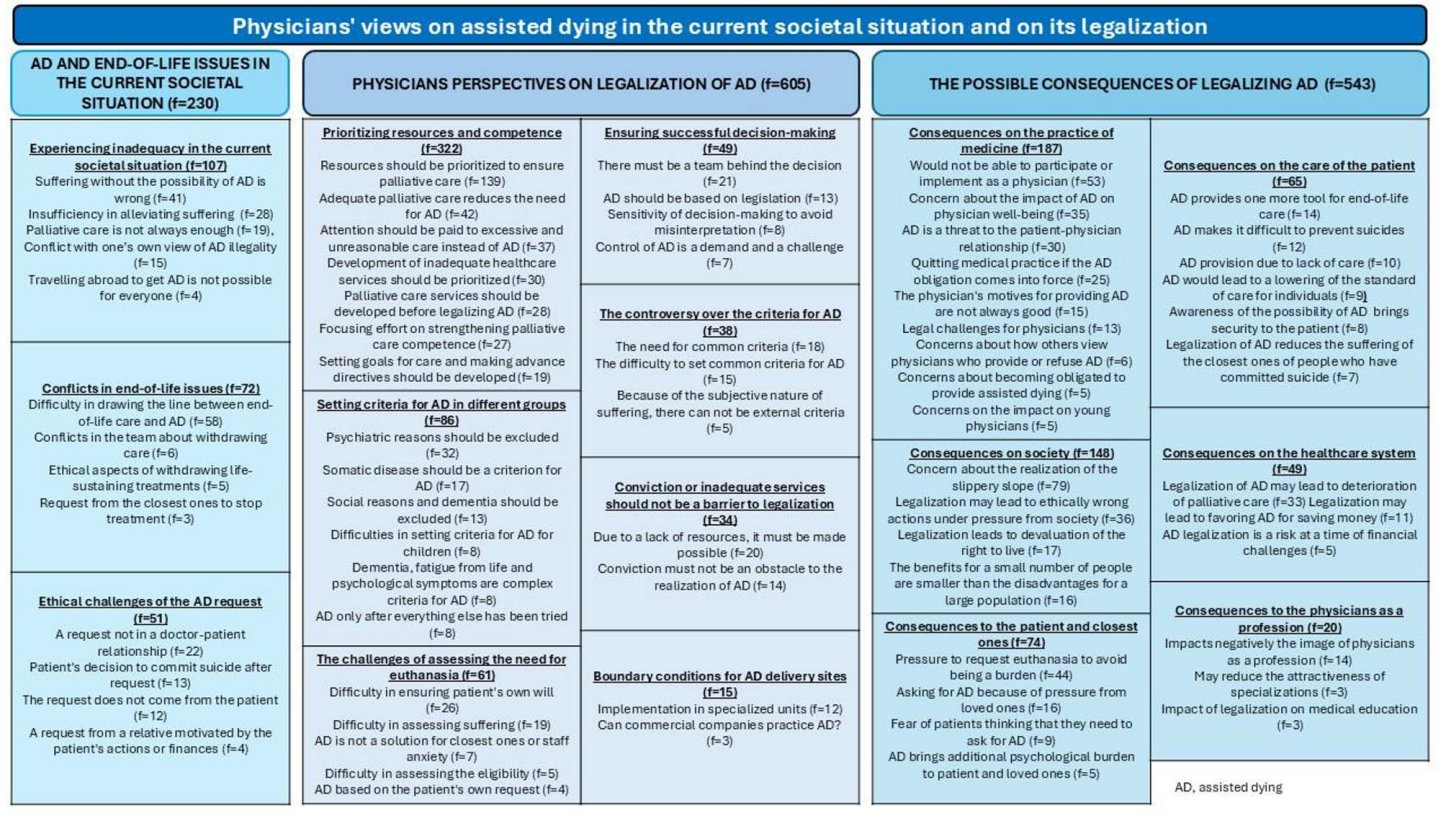
The unified category presented with main categories, categories and sub-categories concerning physicians’ views on assisted dying in the current situation and on its legalization which emerged from the qualitative analysis

**Fig. 2:**
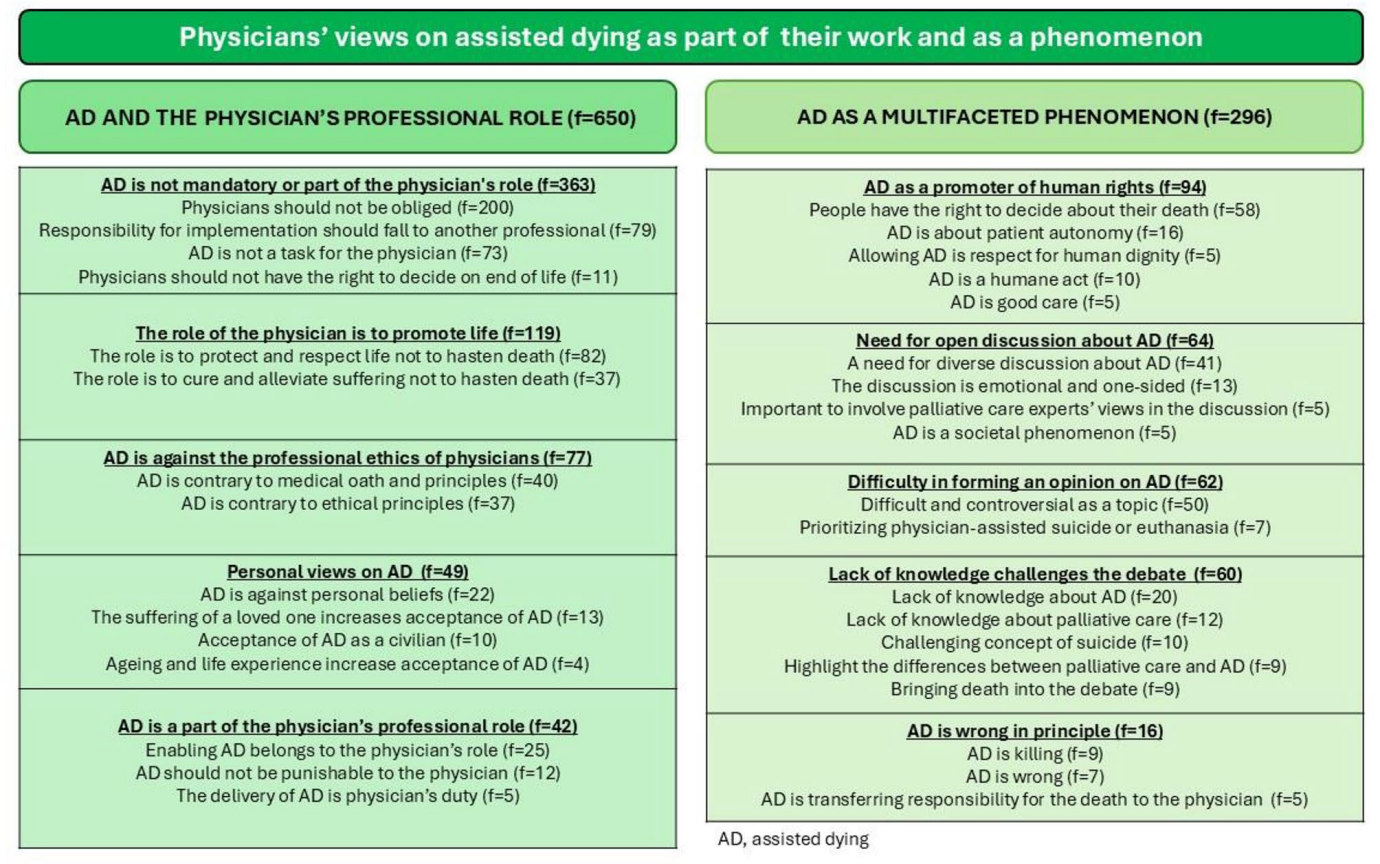
The unified category presented with main categories, categories and sub-categories concerning physicians’ views on assisted dying in the current societal situation and on its legalization, which emerged from the qualitative analysis

From 2565 responders, 3033 answers were received to the open questions. The qualitative analysis yielded two unifying categories. Firstly, ‘Physicians’ views on assisted dying in the current societal situation and on its legalisation’ include three main categories, sixteen categories and seventy-one subcategories (Fig. 1). Secondly, ‘Physicians’ views on assisted dying as part of physicians’ work and as a phenomenon’ include two main categories, 10 categories and thirty-three subcategories (Fig. 2).

### Physicians’ views on assisted dying in the current societal situation and on its legalization

Three main categories were identified, namely, (1) AD and end-of-life issues in the current societal situation (f = 230), (2) physicians’ perspectives on the legalization of AD (f = 605), and (3) the possible consequences of the legalization of AD (f = 543) (Fig. 1).

Assisted dying and end-of-life issues in the current societal situation.

The physicians described feelings of inadequacy in the current societal situation regarding AD and end-of-life situations. Some of them stated that the situation where AD is not legalized puts the patients in a position where they are exposed to unnecessary suffering, which they felt was wrong. Some of the physicians described that they did not have enough possibilities to alleviate suffering in the current situation, due to inexperience, disagreements between the patient, family and physician or lack of resources in healthcare. The physicians stated that palliative care could not always alleviate patients’ suffering due to severe symptoms or difficult disease trajectories. Some physicians described mixed feelings or frustration when they were treating patients or discussing end-of-life care if they were in favor of euthanasia, but it is prohibited. A few physicians stated that the current societal situation is unequal when not all patients have the opportunity to travel to another country to receive assisted death. Examples from the original data:

*634 It is not morally right to keep a person alive when there is no possibility of improving their condition.*

*80Q11 Patient in severe pain and I felt guilt for inexperience and failure to manage pain*

*454 In my work…*,* I have seen that the best possible palliative care is not enough for every situation.*

Physicians described confusion about where the line is between AD and end-of-life decisions, particularly in situations where decisions were made to withdraw care or when treating severe symptoms near death. Withdrawal of care could also cause conflict in the care team. Some physicians described situations where they were confronted by the team about their decisions to withdraw care or where they felt that a colleague was refusing to withdraw care, even though it would have been the right decision. Physicians described that withdrawing care was ethically difficult, particularly in the case of patients who were dependent on ventilatory support and demanded the withdrawal themselves. Some physicians also described situations where the patient’s family requested that the care should be withdrawn, even though the situation did not meet the medical criteria for withdrawal of care. As some of the physicians stated:

*432 Should we talk about assisted suicide if a patient in pain is given so much medicine that the pain is controlled but the risk of fatal respiratory depression is very likely?*

*2936Q11 I cared for a patient close to death. Continued fluids only as long as the veins allowed. The patient passed away. A team member accused me of murder. The relatives did not.*

*4436Q11…parents have asked for withdrawal of treatment in a situation where a long-term prognosis for a child was good.*

The physicians described that they had obtained requests for AD even though it was not legal. Some of them expressed having received requests outside the doctor-patient relationship from relatives, friends, or colleagues. A few physicians described situations which had caused mixed feelings for them, particularly when a patient had committed suicide after having been turned down for a request for AD. Sometimes they described that the request came from family members, not from a patient, and sometimes the motives for these requests were financial or the patient’s misdeeds. Examples from the original data:

*3512Q11 (Relative) in old age once asked for help if/when the time comes that he/she can’t take it anymore. I couldn’t promise and told that it was a very difficult question. I…did not know how to proceed*.

*25Q11 Asked to withdraw care because the patient had already done so many bad things in his life that he should have died.*

Physicians’ perspectives on the legalization of assisted dying.

Many physicians stated that resources should be prioritised for palliative care services, the development of palliative care competence or healthcare services, not AD. Physicians stated that appropriate palliative care reduces or removes the need for AD. Some physicians stated that efforts should be made to avoid excessive and unreasonable care instead of AD and that guidelines and competence should be developed to set goals of care or advance directives instead of promoting AD. Some of them said that palliative care services should be established first, and then the discussion about AD could begin. As some of them stated:

*1051 I believe that euthanasia should not be legalised*,* but that resources should be directed toward better terminal care and palliative care.*

*1027 Instead of discussing euthanasia*,* there should be more discussion (in addition to quality end-of-life care) about goals of care/advance directives*.

*422 Before discussing euthanasia*,* end-of-life care in Finland should be developed to an optimal level throughout the country.*

Physicians described their thoughts on the criteria for implementing AD. On one hand, some argued that patients with psychiatric diseases, dementia, and social suffering should be excluded from AD. On the other hand, others emphasized the complex nature of these situations and stated that these should not be exclusion criteria. Some physicians stated that only suffering from a somatic illness is an acceptable criterion for AD. A few of the physicians brought up the complexity of AD in children. This was described as questions of who can make the decision on behalf of a child and when a child is capable of making a consent decision. Some examples from the original data:

*4433Q11 I think euthanasia should be excluded from psychiatry*,* otherwise the work of psychiatrists becomes impossible. A large proportion of patients want to die in the acute phase.*

*296 It is impossible to set an age limit for ‘eligible to decide’ in a fair way.*

Some of the physicians described that it would be difficult to ensure the patient’s own will and the persistence of the wish of AD when it could be influenced by family, friends, physician or society. They also stated that the degree of suffering was difficult to assess because of its subjective nature. Some physicians stated that sometimes the suffering of the family or staff could influence the patient’s wish for AD. In addition, the patient’s eligibility to decide could be difficult to evaluate. As some of the responders stated:

*725 In either case*,* the patient’s “autonomous wish” may be overwhelmingly influenced by the wishes and judgments or assumptions of relatives or society*,* sometimes even of the doctor*,* so that the patient’s decision is never entirely subjective.*

*959 How would sufficient suffering be defined and who would determine it?*

Physicians described that the decision about AD should be made by a team of physicians or by a multidisciplinary team. Some physicians described that the decision-making process could be challenging when it should be based on successful communication, which can be difficult due to the patient’s condition, language or cultural differences. The need for legislation and systematic control of the AD practice was noted. At the same time, the difficulty of how this could be reliably organised was addressed. Some physicians stated that there should be clear, unambiguous and common criteria before AD could be legalized. In contrast, some of the physicians described the difficulty of establishing clear, unambiguous, common criteria because of the subjective nature of suffering and the unique individual situations of the patients. Some physicians also stated that there should not be external criteria because the decisions should be based on patients’ wishes. As some of them stated:

*558 I think that euthanasia is so susceptible to so many misinterpretations that I cannot imagine as a physician wanting or being able to assess the real motives behind wanting it.*

*467 It is difficult and even impossible for an outsider to assess in which situation the patient’s suffering in the case of a progressive and terminal illness is such that some of the criteria for physician-assisted suicide or euthanasia established by outsiders would be met.*

Some physicians stated that due to the lack of resources in healthcare systems, AD should be legalized to avoid unnecessary suffering of the patients and their families. Some of the physicians stated that conviction and religion should not be an obstacle to the legalization of AD or to performing the act. Some of the physicians also reflected on the constraints of the implementation of AD. It was suggested that there could be a limitation on the number of units and physicians who would have the possibility to perform AD. Some physicians suggested that there should be specific education for physicians about AD. Some physicians also stated that palliative care physicians should be involved in the AD process because of their competence in caring for patients at their end of life. Some physicians also considered whether AD could be practiced in a commercial manner. Examples of the original data:

*225…may allow AD. It is hypocritical to claim that the suffering of people in the final stages can be managed well in the face of all the resource constraints.*

*796 A doctor should not*,* for his own religious reasons*,* deny the willing patients who meet the criteria the right to euthanasia in general.*

*974 I am not in favour of automatically allowing euthanasia in the treatment repertoire of every doctor. It makes more sense for this to be organised in specialised units*,* where people can also be assessed and referred for effective treatment of pain and distress.*

The possible consequences of legalizing assisted dying.

Finnish physicians thought that the legalization of AD could affect the practice of medicine. Some physicians stated that if AD were legalized, they would not be able to participate in the decision-making or the process of administering AD. Physicians hypothesized whether AD would develop in such a way that, even if administering AD was not an obligated task at first, it would sooner or later be mandatory for all physicians. Some physicians stated that if AD were to become mandatory, they would choose to stop practicing medicine. Some reflected about how carrying out AD could harm the mental health of the physician, how it could increase workload and feelings of work exhaustion, and how it would affect young physicians. Some of them described AD as a threat to the physician-patient relationship, as the physician has a dominant position in this relationship, and the patient may lose confidence in the physician’s primary intention to treat the patient. Some physicians also expressed that the physician’s motivation may not always be good due to indifferent attitudes or psychological problems. Some of them stated that legal challenges would inevitably increase with the introduction of AD. Some expressed their views on how AD would affect the attitude of the medical profession toward physicians who perform or refuse to perform AD. Examples of the original data:

*610 I would not be able to actively euthanise another person.*

*364 There would also be a considerable additional psychological burden on the physicians performing/deciding upon euthanasia.*

*513 Physicians are often accused of not wanting to examine or treat because of the need to save money. I believe that legalising euthanasia and taking a positive stance on euthanasia alone would permanently damage the trust in the medical profession and the physician-patient relationship.*

Physicians stated that the legalisation could have an impact on society. Some of them expressed their views about the realization of the slippery slope, meaning that the criteria and boundaries set would expand and could lead to misconduct. Some physicians expressed that the current era might increase social pressure on physicians to carry out AD for the wrong reasons, such as reducing healthcare costs. A few physicians argued that medicine has its historical dark side in the form of violations against humanity and AD could open the path towards new assaults. Some of them thought that the right to life would be compromised and devalued, which could send a wrong message to vulnerable groups. Physicians stated that they understood that some patients could benefit from AD, but the risk to society would be greater, and therefore, the legalization should not be considered. Some examples from the original data:

*500 “Life must have intrinsic value. Euthanasia is a slippery slope*,* as we have seen in other countries and elsewhere. These “side effects” do not become apparent immediately*,* but over years - decades they do.*

*140 At some point*,* politicians will start to take advantage of this legislation in a secularised society.*

The physicians identified possible consequences of the legalization to the patients and their loved ones. Some stated that the legalization could increase the pressure on vulnerable groups of patients to request AD because they could feel like a burden to society or their closest ones. Some of the physicians had experienced that when euthanasia was discussed in the media, some patients had stated their fears that they would need to ask for AD or be offered it as a primary care option. Some physicians stated that AD places an additional psychological burden on the patients and their closest ones when they are in a vulnerable situation. Examples of the original data:

*943 I fear that the acceptance of euthanasia will increase in society and that sick/elderly people may end up in having an unnecessary sense of obligation to leave their environment unburdened. Such a thing is not humane in my opinion.*

*554 At the end of life*,* the patient would have to consider whether he or she would prefer to die a little (or*,* for psychological or other reasons*,* a lot) earlier*,* and this is a very heavy burden to bear for both the patient and the relatives.*

Physicians also commented on the potential impact of the legalization of AD on patient care. Some physicians stated that AD would provide another valuable tool for end-of-life care when a patient’s suffering is unbearable and current care options do not help. Some of them also stated that the knowledge of AD as an option would give patients a sense of confidence and security of care. An option for AD might also reduce the suffering of those closest to a person who, in other circumstances, would commit suicide; if AD were legal, the person could have AD in an acceptable way, which may ease the suffering of those closest to them. On the other hand, some physicians stated that the care of suicidal psychiatric patients could be more difficult. Some physicians stated that insufficient care could be behind the requests for AD. Some of the physicians argued that AD could lead to a situation where the standards of patient care could be lowered because AD could be seen as an easier solution. As some of them stated:

*4357Q11 patients would be happier if euthanasia were an option for them if necessary*

*388 Euthanasia also avoids all the unnecessary and extra emotional suffering that patients’ own*,* often much more shocking*,* suicide decisions cause to their relatives.*

*783 and*,* as I said*,* it would make it even more difficult*,* for example*,* in psychiatry*,* to make patients who want to commit suicide more hopeful towards life.*

The legalization of AD could have an impact on the healthcare services, when some physicians stated that there could be less support for development, research and services in palliative care. Some physicians argued that AD could be used as a substitute for inadequate services. This could lead to the deterioration of the healthcare service system. Some stated that when the government is having financial challenges, it would be risky to push forward the legislation of AD. Some examples from the original data:

*672 I also believe that the acceptance of euthanasia and physician-assisted suicide would undermine the development and research of palliative and end-of-life care.*

*731 In time*,* euthanasia should be allowed*,* but in today’s climate it does not seem safe. (drastic austerity in healthcare*,* performance targets before welfare targets*,* etc.).*

Physicians identified possible consequences of the legalization of AD to their profession. Some of them stated that AD could negatively impact the image of physicians as a profession when the same profession saves and ends patients’ lives. Some physicians also mentioned possible negative consequences on the attractiveness of medical specialities and education. As some physicians stated:

*1112 In principle*,* I see a problem in a situation where the same person is trying to maintain life and actively shorten it at the same time.*

*430 I see a threat that current physicians will not dare to specialise in palliative care,*

### Physicians’ views on assisted dying as part of their work and as a phenomenon

Two main categories were identified, namely, (1) AD and the physician’s professional role and (2) AD as a multifaceted phenomenon (Fig. 2).

Assisted dying and the physician’s professional role

Many physicians stated that if AD were legalized, participation in the process should be voluntary for them. Physicians should not be forced to participate in AD against their will. According to some, AD is not a task which is a natural part of the physician’s role. Others stated that physicians should not have the right to decide when it is time for another person to die. Some suggested that it could be a profession other than physicians who provide AD (for example, lawyers, police, military, a new profession developed for this task or nurses). Examples from the data:

*409 In no case should euthanasia or assisted suicide become an obligation for the physician.*

*1889 It is not and should not be the role of a physician to actively hasten the death of a patient.*

According to many of the responders, the role of the physician is to protect and respect life and cure and alleviate suffering, not to hasten death. Some thought that AD is an act contrary to the medical oath and principles, and also to general ethical principles. As some of them stated:

*913 The physician’s job is to protect life*,* not to get involved in deciding who gets to live and who doesn’t.*

*581 Euthanasia is not treating the patient but eliminating him*,* euthanasia is contrary to the Hippocratic oath of the physician.*

Conversely, some physicians stated that if AD was legalized, enabling AD to suffering patients would be a part of the basic role of the physicians, as they have the competency to ensure a safe procedure. Some stated that it should not be punishable if a physician delivers AD and that it is the duty of the physician to ensure that the patient does not suffer without the possibility of AD. Some examples from the original data:

*474 The physician’s role is to help the person; in some circumstances*,* euthanasia and physician-assisted suicide are also helping the person.*

*388 in the face of an unrelenting and painful death*,* it is the duty of the physician*,* if the patient so desires*,* to help the patient to die. Only physicians have the necessary skills and training to do so.*

Some of the physicians stated that AD was against their personal, religious or other philosophical beliefs. Some stated that they could accept AD as a lay person and had thought that they could accept it for themselves or their closest one if they were suffering. However, they would not accept it as a physician and as part of their practice. Some physicians also identified aspects that influenced their views on AD. When they had experienced end-of-life situations in which their closest ones had suffered, they said that this increased their acceptance of AD. A few physicians stated that ageing and life experience had increased their acceptance of AD.

*532 It is my conviction that the protection of life in all situations and circumstances must be the guiding and sole principle and aim of the medical profession.*

*272. having watched the slow death of a loved one*,* I can’t argue that any amount of medication will guarantee a painless death*,* I cannot in good conscience argue against euthanasia in its entirety.*

Assisted dying as a multifaceted phenomenon

When describing AD as a phenomenon, some physicians stated that AD is the patient’s right as people should have the right to decide about their own death and they have no duty to live if life is a burden or suffering for the person. Some physicians stated that AD supports a patient’s right to autonomy and respects human dignity. Some physicians described that AD would be a human act and a part of good care.

*4447Q11 My opinion is that we should each be able to decide for ourselves whether we want to live or die. If death is the wish*,* it must be faced with dignity and in “civilised conditions”.*

*393 It is then only humane to help a person who is suffering greatly by the last possible means*,* by hastening death.*

For some physicians, AD was wrong in principle. They felt that assisted dying was killing another human being, and some felt that the patients and relatives were trying to shift the responsibility for the wrongdoing to the physician.

*808 Killing another human being is always killing*,* whether it is done legally or criminally.*

*591 I suspect that the request for euthanasia is more about wanting someone else (e.g. a physician) to take over part of the morally unpleasant decision (especially for patients with religious beliefs) than about being incapacitated to kill oneself.*

Physicians identified the need for an open discussion about AD in society. Some physicians stated that the current discussion did not include all necessary stakeholders. They believed that this was important because the topic is a broad societal issue. Some physicians noted that the discussion relied more on emotional aspects than on facts and was perceived as one-sided, which also led to feelings of frustration. Some suggested that palliative care professionals should be involved in the discussion because of their knowledge and experience in end-of-life care. Some stated that the topic felt difficult and controversial and that it was difficult to form an opinion on the subject. Some also stated that more discussions should be focused on whether to prioritize PAS or euthanasia as a better option.

*1931 An important and complex issue*,* both ethically and clinically. It is important to discuss this issue openly*,* objectively and in a pluralistic way so that all those concerned can have their views heard.*

*1103 I think that euthanasia*,* the “good death”*,* is seen in the public debate as too simple. For example*,* if euthanasia could only be requested by a legally competent adult*,* then*,* for example*,* a person with memory loss*,* mental retardation or a terminally ill minor would not be able to request this “good death”.*

*1010 A very difficult question*,* good luck with your reflection. I have personally changed my position many times.*

Lack of knowledge – both among lay people and the medical profession – about AD and end-of-life care issues was seen as a barrier to constructive discussions about AD. Some of the respondents emphasized the importance of highlighting the difference between palliative care and AD in the debates to avoid confusion and fear among laypeople and patients. Some physicians reflected on the use of the term suicide concerning AD and felt that another term would be more appropriate. Others stated that one difficulty in the AD discussion was the medicalization of death.

*954 I have the impression that the media and the general public do not understand the difference between euthanasia and end-of-life care*.

*197 Discussions about death and dying and the taboos surrounding end-of-life care are important in society.*

## Discussion

The results of this study provide new, manifold and broad knowledge about physicians’ views on euthanasia and PAS. The results highlighted that physicians face ethical dilemmas related to a request for assisted dying even though it is not legal in Finland. The phenomenon of assisted dying is seen as multifaceted and controversial. In both qualitative and quantitative results, the dispersion of responders’ opinions of the effect on the physician-patient relationship was seen.

Of the responders, 37% fully/partly agreed that euthanasia would harm the physician-patient relationship, while 30% fully/partly agreed that euthanasia would benefit this relationship. These divided opinions were further emphasized in the qualitative data. The responders were concerned that the physician-patient relationship could be harmed because the trust in the physician’s explicit goal to protect life is threatened if AD is legalized. In contrast, awareness of the possibility of AD was seen as a way to increase patients’ feelings of safety and trust in end-of-life care. In a study made in Canada, medical assistance in dying altered the relationship between patients and families, making it deeper and more personal [22]. Medical care that includes assisted dying was described as relationship-centered care [22]. Building trust in professionals, especially physicians being part of the AD process, was one of the themes that was brought up by patients and families when they were asked how to improve the experiences requesting AD in Canada [41]. The importance of a trustworthy and safe physician-patient relationship seems to be highly emphasized by physicians and patients alike when struggling with the challenging issues of AD.

In our study, responders reflected different criteria for AD. The criteria have been under constant debate since AD practices have been adopted. There is no generally accepted definition of ‘unbearable suffering’ in the context of a request for AD [42]. This leaves a possibility for interpretation, which is also seen in our results. Another debate that was seen in this study concerns the permission of euthanasia and PAS in the case of psychiatric disorders. A systematic review from 2020 showed that articles providing ethical reasoning and opinions in favour of or against assisted death based on psychiatric disorders were evenly distributed [43]. Based on our results, the ambiguity of physicians concerning the criteria for AD remains high.

The conflicts in the care team and confusion about AD and end-of-life questions were particularly related to withdrawal of care and treatment of severe symptoms close to death. Earlier research shows that although clear definitions have been specified for euthanasia and PAS [8], termination of life-sustaining treatments is still confused with euthanasia and PAS, even among physicians [44]. In addition, hastening a patient’s death or a fear of doing so when alleviating severe symptoms or withdrawal of treatment is an ethically challenging question and is sometimes confused with euthanasia or PAS [45]. To support the physician and the care teams, the results call for strengthening the competence on how to deal with end-of-life issues and requests for assisted dying.

In our study, many physicians reflected on physicians’ role in AD. The most frequently merged opinion was that physicians should not be obliged to participate in AD. In most countries where AD is allowed, they have the possibility to refuse based on professional conscience [25]. A few of the responders also stated that they accept AD as a civilian, which further emphasizes the importance of exploring physicians’ role as part of AD and the complexity of this issue.

When reflecting on the results in the light of ethical values, it was clearly seen that physicians’ attitudes toward AD are ambivalent. On one hand, non-maleficence [11, 12] was stated as a justification for not making euthanasia or PAS legal, when life as such was seen to have intrinsic value. In addition, it was stated that the motivation of physicians may not be the patient’s best interests but, for example, saving costs. On the other hand, some responders pointed out that suffering without the possibility of AD causes harm to the patient. When reflecting on beneficence [11, 12], the legalization of AD was justified with the argument that euthanasia and PAS are good care. On the other hand, the legalization was opposed because AD is not considered to be a medical procedure and is wrong as such. In addition, regarding justice [11, 12] the opinions varied as some physicians stated that the resources in healthcare should be allocated to palliative care or the development of other beneficial healthcare services. However, others stated that because of the lack of healthcare resources, euthanasia or PAS should be legalized to ensure equal access to relief of suffering. The legalization of euthanasia and PAS was justified based on the autonomy [11, 12] of the patient and the right to decide when to die. On the opposite side, there were also opinions that the evaluation of an autonomous decision could be difficult. In a previous study, the difficulty of autonomous decision-making was also addressed [46]. It has been argued in previous papers that the pros and cons of AD can be argued with the same ethical values of medicine [47, 48].

This study addressed the complexity of assisted dying as a phenomenon. This is highlighted by the physicians’ various and even opposite opinions, but especially through the difficulty in forming a clear opinion on this challenging issue in the first place. Even though AD is not legal in Finland, ethical dilemmas are obvious in the current societal situation. The physicians expressed general feelings of insecurity and ethical complexities related to end-of-life decisions and even stated that they have faced a request for AD despite the current illegal status of it. Struggling with these challenging issues influenced not only the physicians themselves but also the whole care team. According to a recent editorial in the International Journal of Public Health, guiding and modulating AD’s evolving design and development will be the most urgent medical-ethical challenge for the West in the coming years ahead [49]. Our results confirm this challenge.

### Strengths and limitations

The sample is large and represents well the Finnish physicians [50]. The qualitative analysis process was reported in detail, which increased the reliability of the results. Because of the representative sample, it can be assumed that the study population gave a versatile view of the phenomenon of interest. Furthermore, dependability was strengthened by presenting the figures of all the categories (Figs. 1 and 2). The authenticity was strengthened by providing authentic citations of the data. During the analysis, the researchers constantly discussed the analysis throughout the whole process. Confirmability was strengthened by focusing on the manifest content during the analysis [39].

There are also limitations in this study. Firstly, nonresponse bias might have affected the results, but the number of responders was still substantial. Furthermore, there was no possibility to return the qualitative findings to the physicians for comments [38] due to the anonymity of the responders. It should also be acknowledged that statements and questions on the questionnaire are not without ambiguities, which might have an effect on the answers of participants. Due to the complex nature of AD, it might be impossible to formulate unambiguous statements and questions. There are clear differences in the ethical and practical issues between euthanasia and PAS, but in the open-ended question and some other parts of our results, these two methods of AD were combined. This should be taken into account when interpreting our results.

## Conclusions

This study describes new findings on physicians’ views on assisted dying and its legalization. Assisted dying was seen as a complex issue about which it was difficult to form an unambiguous opinion. Physicians and the care team are faced with ethical dilemmas about topics related to assisted dying, even though it is not legal in Finland. Open, pluralistic discussion, education and recommendations about the professional, ethical and legal aspects of assisted dying and its influence on society and end-of-life care practices are obviously also needed in countries where assisted dying is not legal.

## Electronic supplementary material

Below is the link to the electronic supplementary material.

Supplementary Material 1

Supplementary Material 2

## Data Availability

The datasets generated and/or analyzed during the current study are not publicly available due to the current data policy of Finnish Medical Association but are available from Jukka Vänskä upon justified request.

## Abbreviations

AD: Assisted dying
EAPC: The European Association for Palliative Care
ETENE: The National Advisory Board on Social Welfare and Health Care Ethics
IAHPC: The International Association for Hospice and Palliative Care
PAS: Physician-assisted suicide
WMA: World Medical Association
